# Gene × Environment Interactions in Autism Spectrum Disorders: Role of Epigenetic Mechanisms

**DOI:** 10.3389/fpsyt.2014.00053

**Published:** 2014-08-04

**Authors:** Sylvie Tordjman, Eszter Somogyi, Nathalie Coulon, Solenn Kermarrec, David Cohen, Guillaume Bronsard, Olivier Bonnot, Catherine Weismann-Arcache, Michel Botbol, Bertrand Lauth, Vincent Ginchat, Pierre Roubertoux, Marianne Barburoth, Viviane Kovess, Marie-Maude Geoffray, Jean Xavier

**Affiliations:** ^1^Laboratoire Psychologie de la Perception, Université Paris Descartes, CNRS UMR 8158, Paris, France; ^2^Pôle Hospitalo-Universitaire de Psychiatrie de l’Enfant et de l’Adolescent, Université de Rennes 1, Centre Hospitalier Guillaume Régnier, Rennes, France; ^3^Department of Child and Adolescent Psychiatry, AP-HP, GH Pitié-Salpétrière, CNRS FRE 2987, University Pierre and Marie Curie, Paris, France; ^4^Laboratoire de Santé Publique (EA3279), School of Medicine of La Timone, Marseille, France; ^5^Laboratoire Psychologie et Neurosciences de la Cognition et de l’Affectivité, Université de Rouen, Mont Saint Aignan, France; ^6^Service Hospitalo-Universitaire de Psychiatrie de l’Enfant et de l’Adolescent, Université de Bretagne Occidentale, CHU de Brest, Brest, France; ^7^Department of Child and Adolescent Psychiatry, Landspitali University Hospital, University of Iceland, Reykjavik, Iceland; ^8^Laboratoire de Génétique Médicale, Génomique Fonctionnelle, INSERM U 910, Université d’Aix-Marseille 2, Marseille, France; ^9^Department of Epidemiology and Biostatistics, EHESP School for Public Health, EA 4057 University Paris Descartes, Paris, France; ^10^Service Universitaire de Psychiatrie de l’Enfant et de l’Adolescent Hospitalier Le Vinatier, Bron, France

**Keywords:** autistic spectrum disorders, environment, gene × environment interactions, epigenetics, multifactorial, multidisciplinary

## Abstract

Several studies support currently the hypothesis that autism etiology is based on a polygenic and epistatic model. However, despite advances in epidemiological, molecular and clinical genetics, the genetic risk factors remain difficult to identify, with the exception of a few chromosomal disorders and several single gene disorders associated with an increased risk for autism. Furthermore, several studies suggest a role of environmental factors in autism spectrum disorders (ASD). First, arguments for a genetic contribution to autism, based on updated family and twin studies, are examined. Second, a review of possible prenatal, perinatal, and postnatal environmental risk factors for ASD are presented. Then, the hypotheses are discussed concerning the underlying mechanisms related to a role of environmental factors in the development of ASD in association with genetic factors. In particular, epigenetics as a candidate biological mechanism for gene × environment interactions is considered and the possible role of epigenetic mechanisms reported in genetic disorders associated with ASD is discussed. Furthermore, the example of *in utero* exposure to valproate provides a good illustration of epigenetic mechanisms involved in ASD and innovative therapeutic strategies. Epigenetic remodeling by environmental factors opens new perspectives for a better understanding, prevention, and early therapeutic intervention of ASD.

## Introduction

Biological research in autism has attempted to improve our understanding of the neurobiological mechanisms possibly involved in autistic disorder (AD); studies have been conducted in domains as diverse as genetics, neurochemistry, neuropharmacology, neuroendocrinology, neuroanatomy, brain imaging, and neuroimmunology. For example, structural and functional imaging and neuropathological techniques applied to autism spectrum disorders (ASD) brains have revealed developmental macroscopic and microscopic abnormalities suggesting neuroinflammation in frontal cortex and cerebellar regions, including cytokine production and activation of microglia and astrocytes (1). Studies stress increasingly that AD cannot be summed up or explained by a single biological factor, but rather by a multifactorial etiology. A multidisciplinary biological approach allows us to compare different fields and methodological processes, thus to understand better the neurobiology of autism. However, in spite of the numerous studies conducted on AD during the last decades, it appears that no etiological model, no biological or behavioral marker, and no specific psychopathological process have been clearly identified (negative or contradictory results, associations not replicated). Although the genetic factors and the mode of transmission of AD are not yet fully determined, the underlying genetic architecture, such as known chromosomal rearrangements or single gene disorders, are being identified through, for instance, more and more routine chromosome microarray analysis (CMA) (2). Thus, more than 200 autism susceptibility genes have been identified to date, and complex patterns of inheritance, such as oligogenic heterozygosity, appear to contribute to the etiopathogenesis of autism. Similarly, cytogenetic abnormalities have been reported for almost every chromosome [for a review, see Ref. (3–7) and http://projects.tcag.ca/autism/]. Because of the lack of conclusive results and concensus, it is probably more appropriate to use the concept of *syndrome* to characterize autism. Autism is defined in the ICD-10 and DSM-5 as a delay or abnormal functioning with onset prior 3 years in social communication, and manifestation of restricted, repetitive and stereotyped patterns of behavior, interests, and activities.

Several authors support the hypothesis that the mechanism underlying autism etiology is most likely polygenic and potentially epistatic, and that environmental factors may interact with genetic factors to increase risk (8, 9). Arguments for an environmental contribution to AD come from the growing number of studies on environmental factors in ASD, but also from the current lack of conclusive results on an etiopathological genetic model of autism. It seems important to reframe autism in a multifactorial context. Autism could be considered as a psychopathological organization that would result from the effects of diverse biological factors and/or psychological factors, including genetic factors, environmental factors, and gene × environment interactions. The environmental factors could be post- or prenatal (psychosocial environment but also cytoplasmic and uterine environment, with placental exchanges and hormonal effects).

First, we will examine arguments for a genetic contribution to AD based on updated family and twin studies. Then, after reviewing the possible prenatal, perinatal, and postnatal environmental risk factors for AD, we will discuss the hypotheses concerning the underlying mechanisms related to their role in the development of AD in association with genetic factors. In particular, the possible role of epigenetic mechanisms reported in genetic disorders associated with autism will be considered.

## Genetic Architecture of Autism Risk

Several recent literature reviews underline the important role of genetics in the etiology of AD (10–13). Much of the data come from family and twin studies. The concordance rate among monozygotic (MZ) twins ranges on average from 60 to 90%, and from 0 to 20% among dizygotic (DZ) twins. These rates depend on the diagnosis and on the subtype of autism considered. In addition, they are not sufficient to explain by themselves the autistic syndrome. Autism could be considered as a multifactorial hereditary disorder, in other words a disorder that depends on numerous genes (polygenic heredity) and environmental factors. Although genetic studies have identified hundreds of genes associated with ASD, the exact number remains unknown (10, 14). The wide phenotypic variability of autism may reflect the interaction between genes and environment but also the interaction of multiple genes within an individual’s genome and the existence of distinct genes and gene combinations among those affected.

### Family studies

The prevalence of autism in the general population has been estimated in various ways that depend mainly on sampling methods and diagnostic criteria, as noted already many years ago in the report by Agence Nationale pour le Développement de l’Evaluation Médicale (ANDEM) (15). Thus, the prevalence of autism varies according to the diagnostic criteria of Kanner, DSM-III, and DSM-IV classifications: from 1 to 5/10,000 according to Kanner or DSM-III criteria up to 20/10,000 according to DSM-IV-TR criteria (16). Prevalence of parent-reported diagnosis of ASD among 3- to 17-year-old children in the USA reaches the very high rate of 1/91 (17). Similar results are expected using the DSM-5 criteria given that the diagnosis of autism is only based on ASD in this classification (according to DSM-5 criteria, ASD includes two main domains of autistic behavioral impairments: social communication impairments and stereotyped behaviors or interests). Broadening of the diagnostic criteria for autism and better recognition of the autism behavioral phenotype may explain this rising prevalence, but a true increase in incidence cannot be ruled out [Autism and Developmental Disabilities Monitoring Network, 2008; (18)]. However, Fisch (19, 20) showed clearly that this rising prevalence is related to the use of different diagnostic criteria. He concluded by saying “There is no autism epidemic but a research epidemic on autism.” Still, the reasons of such an increased interest in autism remain to be understood (for example, a better organization of association of parents, more funding contributing to an increase in the number of researchers and studies in autism, a growing interest for social communication impairments in a society promoting social communication networks).

It is noteworthy that there is a male prevalence in autism [about three to four times higher in males than in females (21)], which might also fit with greater social communication difficulties observed in males compared to females with typical development. Studies on the prevalence of autism in families with autistic children show a higher rate than in the general population. The concordance rate for siblings of individuals with autism of unknown cause ranges from 5 to 10% and approaches 35% in families with two or more affected children (22–25). Taken together, the rates of AD in siblings of children with autism are on average 50–150 times higher than the rate of autism in the general population, which suggests that autism has a family feature (family meaning here environmental as much as genetic). Carlier and Roubertoux (26) emphasized that in evaluating the risk, the degree of genetic proximity and the degree of environmental similarity were correlated. Only two studies have attempted to assess the presence of parental pathology at the same time as sibling pathology in the families of autistic individuals. The first was the Utah epidemiological survey (1989) and the second was Gillberg et al. (27) study. Ritvo et al. (25) reported that of the 214 parents seen in the Utah survey, 7 were autistic, the majority being fathers. In the epidemiologically based, case–control study by Gillberg et al. (27) four fathers of the 33 autistic probands were considered to have Asperger’s syndrome. This gives an overall prevalence of autism in parents between the two studies of 2.3%. As underscored by Todd and Hudziak (28) the presence of affected father–son pairs is not compatible with simple X-linked transmission.

### Twin studies

In the field of genetic research on AD, which compares MZ with DZ twins, three interesting results can be presented (see Table 1 for a summary of the results from the updated studies):

- In each study, the concordance rate for MZ twins is higher than for DZ twins.
- The concordance rate of AD in MZ twins is incomplete, suggesting a contribution of environmental factors. Hallmayer et al. (9) underline in their twin studies the involvement of both genetic and environmental factors in the development of ASD.
- In the Folstein and Rutter (29), Bailey et al. (30), and Hallmayer et al. (9) studies, concordance rates vary according to the diagnosis: the concordance rates are higher for the broader autism phenotype than for AD (full diagnostic criteria).

**Table 1:** **Pairwise concordance rates for autism in monozygotic twins (MZ) and dizygotic twins (DZ)**.

Authors	MZ total number of twin pairs	Pairwise concordance rate	DZ total number of twin pairs	Pairwise concordance rate
Folstein and Rutter (29)	11	Autism DSM-III	10	Autism DSM-III
		4 (36%)		0 (0%)
		Cognitive impairment (especially in verbal communication)		Cognitive impairment (especially in verbal communication)
		9 (82%)		1(10%)
Carlier and Roubertoux (26)	30	26 (86%)	9	2 (22%)
Ritvo et al. (31)	23	22 (96%)	17	4 (17%)
Single case studies Smalley et al. (32)	11	9 (82%)	10	1 (10%)
Steffenburg et al. (33)	11	Autism DSM-III-R	10	Autism DSM-III-R
		10 (91%)		0 (0%)
		Cognitive impairment (mainly language/writing/reading impairment)		Cognitive impairment (mainly language/writing/reading impairment)
		10 (91%)		3 (33%)
Bailey et al. (30)	25	Autism DSM-IV	20	Autism DSM-IV
		15 (60%)		0 (0%)
		Cognitive and social impairment		Cognitive and social impairment
		23 (92%)		2 (10%)
Rosenberg et al. (34)	67	Autism spectrum disorders DSM-IV	210	Autism spectrum disorders DSM-IV
		59 (88%)		64 (31%)
Hallmayer et al. (9)		Autism DSM-IV		Autism DSM-IV
	40 Male (M) pairs	17 (43%)	31 MM	4 (13%)
	7 Female (F) pairs	3 (43%)	10 FF	2 (20%)
			55 MF	2 (4%)
		Autism spectrum disorders		Autism spectrum disorders
		DSM-IV-TR		DSM-IV-TR
	45 M pairs	29 (65%)	45 FF	9 (20%)
	9 F pairs	3 (33%)	13 FF	4 (31%)
			80 MF	5 (6%)
Nordenback et al. (35)	13	Autism DSM-IV-TR	23	Autism DSM-IV-TR
		12 (92.3%)		1 (4.3%)

These results point to a possible etiological heterogeneity of autism. The etiology could be different according to the subtype of autism considered, a subtype that could be clinical as much as biological. This may help us to better understand why none of the genetics inheritance models proposed for autism, including the polygenic model, can fully explain the autism phenotype in the family and twin studies presented above. One of the current issues in the field of genetic research on AD is to work on different subtypes in order to identify the relevant genes. There are three main approaches to identifying genetic hotspots or chromosomal regions likely to contain relevant genes: (1) cytogenetic studies, (2) whole genome screens, and (3) evaluation of *a priori* selected candidate genes known to affect brain development or possibly involved in the pathogenesis of autism.

Genome-wide association studies (GWAS) examine associations between disease and genetic variants such as single-nucleotide polymorphisms (SNPs) or copy number variations (CNVs). Genetic variants can be either inherited or caused (which is often the case) by *de novo* mutations. CNVs and SNPs have both been reported to play a major role in autism incidence (36–41). Common SNPs acting additively have been reported as a major source of risk for ASD (42) with heritability exceeding 60% for ASD individuals from multiplex families and approximately 40% for simplex families. CNVs, including insertions, deletions, and repeated sequences, can be highly disruptive to developmentally regulated genes. Several CNV studies (36, 43, 44) identified also structural changes in DNA, which contribute to the risk for ASD. Recent findings suggest the possibility that not only single, but also aggregate molecular genetic risk factors, linked in particular to alterations in calcium-channel signaling, are shared between autism and four other psychiatric disorders (schizophrenia, attention-deficit hyperactivity disorder, bipolar disorder, and major depressive disorder) (45, 46). However, the mechanisms underlying the role of these mutations in the development of ASD phenotypes remain to be ascertained. More generally, children with neurodevelopmental problems, including ASD, are often affected in more than one area of functioning of mental health to the extent that hierarchies of mutually excluding categorical diagnoses have to be considered as conflicting with scientific evidence (47). It suggests, according to Anckarsäter (48), that genetic susceptibilities behind mental health problems have to be sought both in relation to specific problem types and to general dysfunction, using multivariate analyses with measures of all types of mental disorders.

Concerning candidate genes, several of them have been studied at chromosome regions 7q22–q33 or 15q11–q13, and variant alleles of the serotonin transporter gene at 17q11–q12 are more frequent in individuals with autism [see Ref. (12), for a review]. Linkage data from genome screens and animal models suggest also a possible role of the oxytocin receptor gene at 3p25–p26 (49). Interestingly, the majority of the genes reported to be associated with autism is involved in various physiological processes, such as chromatin remodeling, metabolism, translation, and synaptogenesis. These genes may converge into pathways affecting distinct neuronal functions such as synaptic homeostasis. Such a genetic basis of synaptic and neuronal signaling dysfunction in ASD has been confirmed by recent findings (50) demonstrating differences in transcriptome organization between autistic and normal brain through gene co-expression network analysis.

Finally, it should be highlighted that the polygenic model does not exclude a role of environment. It is noteworthy that heritability (*h^2^*) is defined as *h^2^* = GV/(GV + EV) where GV is the cumulative genetic variance and EV, the environmental variance (51). The possible prenatal, perinatal, and postnatal environmental risk factors for ASD are presented below.

## Prenatal, Perinatal, and Postnatal Environmental Risk Factors for ASD

The prenatal factors associated with autism risk in the meta-analysis provided by Gardener et al. (52) were advanced paternal and maternal age at birth, gestational diabetes, gestational bleeding, multiple birth, being first born compared to being third or after, and maternal birth abroad. In fact, several recent studies suggest that parental immigration, especially maternal immigration but also paternal immigration, is a risk factor for ASD (53–59). This association between migration and autism is more particularly observed in male children of immigrant parents living in urban areas compared to rural areas (60). In addition, concerning the prenatal risk factors for AD, a rare consistent association with AD is *in utero* exposure to two known teratogenic medications, thalidomide, and valproate (valproate is a broad-spectrum anticonvulsant drug used in seizures, bipolar disorder, or migraine headache), or the abortifactant misoprostol (7, 61–63). Thus, children exposed to valproate *in utero* were seven times more likely to develop autism than those not exposed to antiepileptic drugs (61, 62). A large population-based cohort study of all children born alive in Denmark from 1996 to 2006 was conducted on 655,615 children, including 508 prenatally exposed to valproate and 5437 identified with autism spectrum disorder (2067 with AD). Children of women who used a high valproate dose (>750 mg/day) or a low valproate dose (<750 mg/day) early (first trimester) or later in pregnancy had significantly a higher risk of ASD and AD compared with children of women who did not use valproate (even after adjusting for maternal epilepsy and parental psychiatric history, or restricting the analysis to children without congenital malformations), whereas this increased risk was not observed for other antiepileptic drugs used as monotherapy. The mechanisms of action of valproate will be developed later in the next section. Furthermore, prenatal exposure to folic acid (known to decrease the risk of neural tube defects) has also been associated with the risk of autism (64). As folate and folic acid are essential for basic cellular processes (including DNA replication as well as DNA, RNA, and protein methylation), it could not be excluded that, depending on timing and dose, such nutritional supplements might also have adverse effects. Also, the time period at which folic acid was added to the diet of women of childbearing age coincides with the apparent onset of a continuous increase in the prevalence of autism. However, a recent well-controlled epidemiological study (65) disconfirms this claim and reports, as underlined by Berry et al. (66) or Vahabzadeh and McDougle (67), a lower incidence of AD in children whose mothers received prenatal folic acid supplementation around the time of conception (64/61 042 or 0.10%) than in children whose mothers did not take folic acid (50/24 134 or 0.21%). Similarly, in children from countries without folic acid supplementation, autism has been linked to two polymorphisms of the methylenetetrahydrofolate reductase gene (MTHFR), which is essential for DNA biosynthesis and the epigenetic process of DNA methylation (68). Although such findings do not establish a causal relation between folic acid use and a lower incidence of AD, they do provide an impetus for further study. Finally, some authors (69, 70) have suggested a low but possible risk of neurological problems and imprinting disorders (such as Beckwith–Wiedemann syndrome and Angelman syndrome which are genetic disorders associated with autism) in children conceived by *in vitro* fertilization (IVF). However, earlier investigations on possible links between assisted reproductive technologies (ART) and autism have shown inconsistent results (71, 72). More recent epidemiological studies involving larger populations show that IVF is not associated with autism (73), but rather with a small increased risk of intellectual disability (74). According to Sandin et al. (74), the risk for ASD was significantly higher following intra-cytoplasmic spermatozoid injection (ICSI) using surgically extracted sperm and fresh embryos compared to IVF without ICSI with fresh embryo transfer.

During the perinatal period, Guinchat et al. (75) pointed to three factors associated positively with the development of AD: prematurity (the risk for autism increased with the severity of preterm birth), abnormal presentation in general and breech presentation in particular, and planned cesarean section. However, the role of cesarean section as an independent risk factor for AD needs to be clarified given that breech presentation is a common cause of first cesarean delivery. Thus, Bilder et al. (76) reported that after correction for breech presentation, the observed association between cesarean section and AD lost statistical significance. Other studies grouped cesarean section in one specific variable [for a review, see Ref. (75, 77, 78)], but cesarean section emerged as an independent risk factor only in the Hultman et al. (79) study. During the neonatal period, conditions potentially related to hypoxia, such as umbilical-cord complications, low 5-min Apgar score, being small for gestational age, low birth weight (especially when <1500 g), fetal distress, or meconium aspiration, as well as birth injury or trauma, summer birth, feeding difficulties, neonatal anemia, ABO or Rh incompatibility, and hyperbilirubinemia were significantly (*P* < 0.05) associated with autism [for a review, see Ref. (8, 75)]. Thus, Maimburg et al. (80, 81) and Buchmayer et al. (82) published large population-based studies associating hyperbilirubinemia with independent risks for AD that might be related to the potential toxicity of hyperbilirubinemia on basal ganglia and cerebellum. It is noteworthy that prematurity might be a variable masking the effect of hyperbilirubinemia. Interestingly, summer season [summer birth was significantly associated with an elevated risk of autism, RR: 1.14, *P* = 0.02; (8)] corresponds to the longest days of the year. It strengthens the hypothesis developed by several authors [for a review, see Ref. (83–85)] of a possible role of a deficit in melatonin in the development of ASD (the production of melatonin is powerfully suppressed by light acting through the retino-hypothalamic tract). Finally, the perinatal factors with the strongest evidence against a role in autism risk included anesthesia use during delivery, assisted vaginal delivery, post-term birth, high birth weight, or head circumference (8).

Effects of exposure to air pollution during pregnancy in the first year of life deserve particular attention, especially because they might be mediated by epigenetic mechanisms as in valproate exposure. Epidemiological studies (86, 87) reported associations between autism and air pollution at the birth and early life residences. Thus, residential proximity to freeways in California within 309 m during the third trimester of pregnancy and at birth was found associated with a risk of ASD about twofold higher (88). Studies in animal models (rodents) and humans described developmental effects of air pollution following prenatal and early life exposure, such as altered neuronal differentiation, impaired cognitive functions, and white matter abnormalities (89–91). Given the male prevalence observed in autism, it is noteworthy that adult male mice but not females, showed increased depression-like responses and low resilience to stress in the tail suspension test following prenatal exposure to urban freeway nanoparticulate matter. In this line, Volk et al. (92) found that exposure during pregnancy and the first year of life to traffic-related air pollution was associated with autism (DSM-IV and ICD-10 criteria based on the ADI-R and ADOS scales). Children residing in homes with the highest levels of modeled air pollution (>31.8 ppb) were three times as likely to have autism compared to children residing in homes with the lowest levels of exposure (<9.7 ppb). An increasing probability of autism was seen with increasing air pollution (nitrogen dioxide and particulate matter less than 2.5 and 10 μm in diameter: PM_2.5_ and PM_10_) with a plateau reached at a threshold above 25–30 ppb. Associations were reported for each trimester of pregnancy but the smallest magnitude of the effects was observed for the first trimester. Neurodevelopmental effects of prenatal and/or early life exposure to polycyclic aromatic hydrocarbons may be mediated by epigenetic effects (93). However, the results could also be affected by unmeasured confounding factors associated with both autism and exposure to traffic-related air pollution.

Furthermore, maternal depression (prenatal but also postnatal depression given that it is in fact very difficult to dissociate depression during pregnancy from perinatal/postnatal depression) raises an interesting issue with regard to risk factors for ASD. Interestingly, common genetic factors contributing to depression and autism have been reported (45, 46). A study (94) conducted on 4429 cases of ASD (1828 with and 2601 without intellectual disability; antidepressant use during pregnancy for 1679 cases) showed that a history of depression during pregnancy but not paternal depression was associated with an approximately 60% increase in risk of ASD in offspring (raw odds ratio 1.61, 95% confidence interval 1.17–2.23, *P* = 0.004), particularly without intellectual disability (adjusted odds ratio 1.86, 95% confidence interval 1.25–2.77, *P* = 0.002), and more precisely for mothers reporting antidepressant use during pregnancy but independently of the type of antidepressant (adjusted odds ratio when depression with antidepressant use 3.34, 1.50–7.47, *P* = 0.003; adjusted odds ratio in case of depression without antidepressant use 1.06, 0.68–1.66, not significant). These results are in line with the Croen et al. (95) study reporting association between use of selective reuptake inhibitor (SSRI) antidepressants during pregnancy and ASD in offspring. However, antidepressant use during pregnancy explained only 0.6% of the 4429 cases of ASD in the Rai et al. (94) study. The authors conclude that assuming causality, antidepressant use during pregnancy is unlikely to have contributed significantly toward the observed prevalence of ASD as it explained less than 1% of the cases. In summary, the Croen et al. (95) study suggests an effect of antidepressant use during pregnancy but, as supported by the Rai et al. (94) study, a risk for ASD in severe maternal depression during pregnancy cannot be excluded or reduced to the effect of antidepressant use. Further studies are requested to test fully these hypotheses through a scientific approach and methodology. It is noteworthy that to avoid the possibility of reverse causality reported by Daniels et al. (96), only diagnoses of maternal depression recorded before birth were considered by Rai et al. (94). Also, two meta-analyses (52, 97) underlined the lack of studies with psychiatric diagnosis of parents before the birth of children with autism, suggesting that depression during pregnancy may be underestimated. Finally, given the continuity between prenatal and postnatal depression, an effect of maternal postnatal depression on early infant development cannot be ruled out.

It is difficult to establish if these prenatal, perinatal, and neonatal factors are independent environmental risk factors for ASD involved in cause–effect relationship or are associated with ASD but result themselves from other factors that still need to be identified. Thus, maternal immigration is probably not an independent environmental risk factor for ASD but might be related to other factors such as prenatal and/or postnatal maternal depression. Similarly, several potential risk factors for ASD that occur during the neonatal period, such as low Apgar scores or birth weight, shortened gestational age or even breech presentation, may be the consequence of other risk factors during the prenatal and perinatal periods, such as prematurity. Furthermore, the possible effects mentioned previously of prematurity or severe maternal depression on the risk for ASD could be related, for example, to a dysfunction in brain development (e.g., prenatal depression is associated with a modified biological profile involving, in particular, the serotoninergic system and the hypothalamo-pituitary adrenal axis) and/or a deficit in very early social interaction and sensory stimulation. Finally, it is noteworthy that in Gardener et al. (8) meta-analysis, overall, preterm birth was not associated with the risk of autism, although there was significant heterogeneity across studies.

Concerning psychosocial factors occurring during the postnatal period, such as a severe deficit in very early social interaction, AD has been reported in children institutionally exposed to profound early social deprivation (98–100). However, several issues have to be raised. First, if the pattern of autistic-like behavior found by Rutter et al. (99) at 4 years of age in the sample of children from Romanian institutions was indistinguishable from that seen in typical children with autism, by age 6 years this “quasi-autistic” pattern had diminished and a number of atypical features were observed: the children displayed more flexibility in communication and a substantial social approach up to, for some of them, an indiscriminately overfriendliness that is usually associated with disinhibited attachment. Furthermore, the follow-up of 28 children with “quasi-autism” from Romanian institutions, using the autism diagnostic interview-revised [ADI-R; (101)] showed that by age 11–12 years, just over 1 out of 10 children who experienced profound institutional deprivation still displayed a quasi-autistic pattern but a quarter of the children lost their autistic-like features, and dishinibited attachment with poor peer relationships was also observed in over half of these children (100). It is noteworthy that most of the children with quasi-autism had an IQ in the normal range. Finally, methodological issues can be raised concerning the Rutter et al. follow-up that did not study the relationship between the presence of autistic-like features and the duration of the profound institutional deprivation or the age of the children when they experienced this social deprivation.

Concerning neurophysiological factors occurring during the postnatal period, and more specifically severe sensory deprivation, several studies report a high risk for AD in congenitally severely visually impaired children. Thus, AD was observed in 10 out of 24 (42%) congenitally blind children (102) and autism was also reported in 30 out of 157 (19%) children with varying ophthalmological problems (103). In addition, only 31% of Swedish children with AD (*n* = 45) were found to have normal visual acuity (104). Similarly, several studies have reported abnormally high rates of AD in hearing impaired individuals [from 1.7 up to 7%; (105, 106)]. Inversely, hearing problems, including hearing loss, were described in 10% of Swedish children and adolescents with autism (*n* = 199) (107). Furthermore, very high rates of ASD have been observed in congenital conditions (up to 68% in CHARGE syndrome, 45% in Möbius sequence, 42% in oculo-auriculo-vertebral spectrum) involving multiple sensory deficits such as impaired vision associated with reduced hearing. These study groups and case reports suggest that severe multimodal sensory deprivation is a good candidate risk factor for AD. Genetic disorders such as CHARGE syndrome support the hypothesis that genetic factors could lead to multiple simultaneous sensory deficit occurring at a critical period of development that, in turn, would play a role in the pathogenesis of AD. Also, genetic disorders such as CHARGE syndrome support the hypothesis of common genetic risk factors for ASD given that CHARGE syndrome is due to mutations in CHD7, which is a homolog of CHD8, one of the most recurrently affected genes in autism cohorts (108). Taken together, these studies suggest that impairment in cross-modal sensory perception (impaired vision associated with reduced hearing) contributes more to the development of AD than impaired vision alone, which appears to be a more important risk factor for AD than reduced hearing alone. This is in line with a model, which postulates that AD would be related to impairment in the developmental sequence involving cross-modal sensory perception, body-image construction, sense of body self, self/non-self differentiation and in turn development of social communication (109). However, it is noteworthy that CHARGE syndrome, Möbius sequence, and oculo-auriculo-vertebral spectrum are associated with extended developmental defects that might be involved in the risk for ASD. Thus, CHARGE syndrome includes multiple malformations (see Table 2), Möbius sequence is associated with brainstem hypoplasia (110), and cerebral abnormalities are observed in oculo-auriculo-vertebral spectrum (111). Finally, another limitation is related to the assessment of ASD in congenital conditions due to the difficulty to use current autism diagnostic instruments in individuals with intellectual disability, cranial nerve palsies (most commonly affecting the facial nerve), and sensory deficits (patients often being severely visually impaired or blind, hearing impaired or deaf).

**Table 2:** **Genetic disorders with epigenetic mechanisms associated with autistic syndrome**.

Genetic disorder	Estimated rate (%) of the disorder in autism	Estimated rate (%) of autism in the disorder	Degree of intellectual disability	Autistic behaviors	Other behaviors	Other symptoms
CHARGE syndrome (CHD7, 8q21.1) (112–116)	<1	15–68	Variable (normal to severe) but often normal IQ	Severe autistic syndrome to Asperger syndrome	Hyperactivity, obsessions and compulsion, tic disorders	Coloboma of the eye, heart defects, atresia of the nasal choane, retardation of growth, and/or development, genital/urinary abnormalities, ear abnormalities/deafness
Maternal 15q11–q13 duplication (117–119)	1–2	80–100	Severe	Severe autistic syndrome with severe expressive language impairment	Hyperactivity, anxiety, tantrums, and aggression	Facial dysmorphism, seizures (75%), hypotonia, genitor/urinary abnormalities
Angelman syndrome (maternal 15q11–q13 deletion, paternal uniparental disomy) (118, 120, 121)	1	80–100	Severe	No language, stereotyped behaviors, immutability	Attention deficit with hyperactivity disorder (ADHD), paroxysmal laughter, tantrums	Facial dysmorphism, microcephaly, seizures (>1 year), ataxy, walking disturbance
Prader–Willi syndrome (maternal 15q11–q13 disomy, paternal deletion) (122–124)	–	–	Mild	Motor and verbal stereotypies, rituals	Hyperphagy, obsessions and compulsions, tantrums	Obesity, growth delay and hypogonadism, facial dysmorphism
Fragile X syndrome*(FXS, Xq27.3) (125–128)	2–8	10–33	Variable	Poor eye contact, social anxiety, language impairment, stereotyped behaviors	Hyperactivity with attention deficit, sensory hyper-reactivity	Facial dysmorphism, macro-orchidism
Rett syndrome (MECP2, Xq28) (129–131)	<1 in female	80–100	Severe	Stereotyped hand movements, absence of language, loss of social engagement	Stagnation stage (6–18 months) in girls, then regression stage (12–36 months), pseudostationary stage (2–10 years), and late motor deterioration (>10 years)	Head growth deceleration, progressive motor neuron (gait and truncal apraxia, ataxia, decreasing mobility) and respiratory (apnea, hyperventilation, breath holding) symptoms
Down syndrome (Trisomy 21) (132–134)	2,5	05/10/14	Variable but usually severe	Severe autistic syndrome	–	Facial dysmorphism, heart and intestine malformations
Turner syndrome(135)	–	3	Usually normal IQ	Females monosomic for the maternal chromosome × score significantly worse on social adjustment and verbal skills	–	Short stature, skeletal abnormalities, absence of ovarian function, webbed neck, lymphedema in hands and feet, heart defects and kidney problems
Phelan–McDermid syndrome (PMDS) (136)	–	75–84	Severe to profound	Variable autistic syndrome: from autistic disorder to autism spectrum disorders (ASD) including delayed or absent verbal language	Global developmental delay	Dysmorphic features, hypotonia, gait disturbance, recurring upper respiratory tract infections, gastroesophageal reflux and seizures
Beckwith–Wiedemann syndrome (BWS) (137)	–	6.8**	Usually normal IQ	ASD	–	Pre- and postnatal overgrowth (hemihyperplasia, macroglossia, visceromegaly) and increased risk of embryonal tumors
Williams–Beuren syndrome (WBS) (7q11.23 deletion) (138, 139, 216, 217)	<1	<5	Mild to moderate	Autistic syndrome (from social withdrawal and absence of verbal language to overfriendliness and excessive talkativeness)	Visual spatial deficit, hyperacusis, feeding and sleep problems	Facial dysmorphism, short stature, heart and endocrine malformations, hypercalcemia

*The estimated rate of FXS in individuals diagnosed with autistic disorder varies, according to Harris et al. (128) from 2 to 8% (in fact from 0 to 8% according to the studies), and the estimated rate of autism among FXS individuals varies from 10 to 33% (140).

**A diagnosis of ASD was reported for 6 out of 87 children with BWS based on parental questionnaires (137). This result requires replication using valid diagnostic assessment tools.

In conclusion of this descriptive part on the prenatal, perinatal, and postnatal risk factors, it appears that no specific factor has been identified and no individual factor has been consistently validated as an independent environmental risk factor for ASD. This suggests that future research on environmental risk factors for AD, rather than focusing on a single factor, should study a combination of factors through an integrated approach including gene × environment interactions and conduct multivariate analyses.

## Hypotheses on the Role of Environmental Factors in Association with Genetic Factors

First, “the epiphenomenon hypothesis” argues for a primary role of the genetic susceptibility to autism and proposes that genetic factors increase the risk for both autism and the associated prenatal, perinatal, and postnatal complications. This hypothesis is supported by Glasson et al. (141).

A second hypothesis, “the heterogeneity hypothesis,” proposes that the contribution of genetic and/or environmental factors varies according to the cases. AD might be due mainly to genetic factors in some cases [for example, neonatal congenital malformations are significantly associated with an increased risk for autism; (8)] or mainly to environmental factors in other cases (for example, *in utero* exposure to valproate) with possible cumulative effects mediated by different environmental factors and/or genetic factors (for example, see the CHARGE syndrome in Table 2). Some authors (142–144) suggest that the greater the genetic contribution, the more dysmorphic signs, and cognitive impairment are present. Thus, children with AD showing a higher number of minor physical anomalies have lower IQ and are more at risk for genetic variations (142). The finding that unbalanced chromosome abnormalities are found predominantly in children with autism who are dysmorphic (142, 143, 145), strengthens this hypothesis. It is noteworthy that large chromosomal abnormalities are more often found in children with dysmorphic features, whereas smaller CNVs involving the same region and *de novo* single-nucleotide variants of major effect are found often in individuals without dysmorphic features. However, there is no evidence that the role of environment is more important in the case of absence of *de novo* event, as common variants were shown to contribute substantially to autism risk (42). The debate does not focus anymore on a possible contribution of genetic factors to the risk for autism but on the magnitude of this contribution. Contemporary research efforts are moving away from the search for a condition-specific genetic factor to embrace a more cumulative model based on elevated risk as a function of smaller gene point mutations. Interestingly, the hypothesis, mentioned previously, related to genes altering the synaptic homeostasis leads to the perspective of possible autism phenotype reversals (146) and raises issues concerning a possible role of environmental factors associated with genetic factors. Indeed, it has been shown in mouse models of autism that certain neuronal defects can be reversed in the mature mouse brain, either by restoring the gene function, decreasing mRNA translation, or modulating the balance between excitation and inhibition. The early signs of autism are in fact still largely unknown, which hints that during this premorbid period, there might be a discrete window for reversing the pathological process. This window of development could correspond to early critical periods when brain development is particularly sensitive to experience and when brain plasticity, involving sensory systems but also motor functions and cognition, is possible (147). After these critical periods, the level of plasticity is reduced due to the development of myelin and perineuronal networks that drastically prune neuronal outgrowth in the mature brain and lead to functional modifications or fine-tuning in the excitation–inhibition balance (148).

Finally, another hypothesis is to consider the genetic pre-disposition to AD as resulting from different chromosomal or gene variations and to propose that environmental factors associated with these genetic factors would modify the phenotypic expression of AD and lead to a similar clinical phenotype. The different genetic disorders associated with autistic syndrome might share the same environmental factors that could contribute to the expression of behavioral autistic impairments. For example, oxidative stress might be a candidate mechanism linking genetic and environmental factors in genetic disorders associated with autism (149–151). Thus, regarding the possible molecular mechanisms that might be shared in autism and fragile X syndrome (FXS), it has been proposed that increased oxidative stress in the brain might be a common factor. The loss of *FMR1* expression in FXS leads to the absence of fragile X mental retardation protein (FMRP), which is primarily involved in binding mRNAs. The absence of FMRP, as shown in *FMR1*-knockout mice, leads to increased oxidative stress (152–154). It can be hypothesized that any chromosomal or gene variations leading to increased oxidative stress would contribute to the expression of a behavioral autistic phenotype. Inversely, heterogeneity of environmental factors may lead to clinical subgroups with different cognitive–behavioral phenotypes. Lacaria et al. (155) developed a mouse model for Potocki–Lupski syndrome (PTLS) (PTLS is a genetic disorder associated with autism and caused by a 3.7-Mb duplication in 17p11.2), and rearing this animal model in an enriched environment (enriched cages were larger and contained enrichment items such as increased number of mice per cage to enhance social behavior) mitigated the autistic-like abnormal behavioral phenotypes, suggesting a role for gene–environment interactions in the determination of CNV-mediated autism severity. The authors suggest a potential link between the behavioral benefits of environmental enrichment and the underlying changes observed in their study for the serotoninergic and dopaminergic pathways. This is particularly interesting with regard to the abnormalities of the serotoninergic and dopaminergic systems reported in autism [for a review, see Nakamura et al. (156)]. Furthermore, epigenetics is a good illustration of the effects of environmental factors on gene expression. Epigenetics can be viewed as a candidate biological mechanism that is a part of the more general hypothesis of “gene × environment interactions.” Environmental factors/events can occur during early life and be involved in the regulation of neural development associated with synaptic plasticity even at later developmental stages (157). This hypothesis states that environmental factors interact with genetic factors that would not or less influence development otherwise. Gene × environment studies are needed to test this hypothesis and raise the issue of power. The issue of power refers to the possibility to detect an effect based on sample size. Indeed, interaction effects, such as gene × environment ones, require large samples and high power in order to detect the effects as these effects are often small but perhaps truly present and worth integrating in models. Furthermore, interaction effects are known to be difficult to observe because measurement errors or random noise associated with the variables involved in the interaction cumulate, thus “hiding” interaction effects unless they are very strong. The use of large samples reduces the tendency for noise in the data to be an issue, as errors tend to cancel each other out in a large sample, thereby increasing the ability to see an interaction effect.

More precisely, *epigenetics* refers to functionally relevant modifications to the genome that influence gene expression without involving a change in nucleotide sequence. Epigenetic modifications include DNA methylation and various modifications (e.g., methylation, acetylation) of histone proteins that are complexed with DNA to form the chromatin. Epigenetic modifications of histone proteins are generally transient and reversible, whereas epigenetic modifications of DNA are usually more stable. It is noteworthy that environmental events involved in epigenetic mechanisms may be, as underlined by Bagot and Meaney (157), internal or external to the organism (e.g., changes in the availability of glucose, electrical impulses, or social interaction and maternal care). Thus, maternal care such as pup licking/grooming from the mother over the first week of postnatal life provides tactile stimulation for the neonate, which increases hippocampal glucocorticoid receptor gene expression through epigenetic modifications of DNA and decreases HPA responses to stress (158, 159). Inversely, manipulations imposed on the mother that decrease pup licking/grooming such as chronic stressors, are associated with decreased hippocampal glucocorticoid receptor expression, increased hypothalamic expression of CRF, and enhanced behavioral and HPA responses to stress (160, 161). According to Bagot and Meaney (157), these findings suggest that maternal care can stably affect gene expression that in turn mediates the expression of individual differences in behavioral and neuroendocrine responses to stress in adulthood. Futhermore, epigenetic modifications of DNA are stable and can also be transmitted across generations. Indeed, early-life postnatal adversity in animal models can persistently affect behavior across generations and DNA methylation in the germline. Thus, chronic and unpredictable early maternal separation from day 1 to 14 induces in mice depressive-like behaviors and DNA methylation changes in the separated male pups (this finding was not observed in female pups confirming previous findings that maternal separation and prenatal stress have a negative influence primarily in males [162]), but also in female offspring of males subjected to maternal separation, despite the fact that these males were reared normally (163). The authors (163) showed that chronic and unpredicatble maternal separation alters DNA methylation in the promoter of several genes in the germline of the separated males and these changes are transmitted across several generations through a complex and sex-specific mode (transmission occurs through males by epigenetic germline inheritance and affects the offspring in a sex-dependent manner).

Environmental events occurring during early development can activate cellular signaling pathways associated with synaptic plasticity even at later stages in development. The critical period could be during fetal development [as suggested by *in utero* exposure to valproate; (164)] but also during early-postnatal development [as suggested by studies in animal models concerning the first weeks of postnatal life (157)]. In animal studies, autistic behaviors have been observed following administration of valproate during prenatal life or during weaning (stereotyped behaviors, social interaction deficit, self-injurious behaviors, enhanced anxiety as well as impaired cognitive, motor, and attention development) (165–167). In addition, Rodier’s group exposed rat embryos to valproate at the period of neural tube closure and it led to a reduction of the vermal posterior lobe (168). This study, showing that early chemical exposure can provoke late developmental cerebellar anomalies is of interest but the face validity and construct or etiological validity of this model of autism are questionable considering that animal behaviors have not been studied and cerebellar vermian anomalies reported in individuals with autism are controversial. In fact, several authors have proposed exposure to valproate as a possible neurobehavioral model of autism (169–172). However, as underlined in our review on animal models relevant to autism and schizophrenia (173), the main interest of animal models is not to validate a specific categorical model of autism, but rather to study behavioral and neurobiological mechanisms possibly involved in ASD through a multidimensional approach. Concerning its mechanisms of action, valproate has known epigenetic effects. Phiel et al. (174), using cell lines, showed that valproate at therapeutic concentrations inhibits histone deacetylases; this inhibition correlates with increased expression of multiple genes. Milutinovic et al. (175) reported that valproate affects not only histone modification and gene expression, but also DNA de-methylation in human embryonic kidney cell lines, linking epigenetic modifications of both histones and DNA to gene expression.

Oh et al. (176) studied effects of other environmental factors on DNA methylation, using a mouse model of maternal adversity – based on a deficit in the maternal 5-HT_1A_ receptor [reduced binding of 5-HT_1A_ has been found in peri/postpartum depression, a condition that can represent early life adversity for the offspring (177)], which causes innate anxiety, increased stress reactivity, and impaired vocal communication in the offspring – and genome-wide DNA methylation analyses. One rationale for this study was that adverse gestational and postpartum maternal environment might be a contributing factor in the development of autism based on autistic behaviors following prenatal exposure to valproate (as mentioned above), mental disorders associated with parental adversities during early-postnatal life (178), and also effects of deficit in maternal care on increased stress responses in the offspring (157). The authors found that adverse maternal environment induced DNA methylation changes in the offspring and the affected genes encode proteins involved in synapse formation and function (reduced expression of cell adhesion and neurotransmitter receptor genes was observed). They conclude that the differential methylation expression of a large number of pre- and post-synaptic cell adhesion molecules and neurotransmitter receptor genes all involved in neuronal excitability as well as anxiety, suggests a wide-ranging and permanent epigenetic effect of the adverse maternal environment on synaptic plasticity and neuronal excitability. It is noteworthy that mutations in some of these genes such as genes encoding cadherins and neurexin–neuroligins in human, which were differentially methylated following exposure to adverse maternal environment in mice, have been associated with ASD (179–182). A limitation of this animal model is that it does not offer a model of autism, but rather a model of 5-HT_1A_ receptor-deficient maternal environment. However, studying the effect of 5-HT_1A_ receptor-deficient maternal environment on DNA methylation as well as on anxiety-like behavior and impaired vocal communication in the offspring is of interest in autism, especially given central and peripheral alterations in serotonin in autism (156, 183).

Concerning epigenetic histone modifications possibly involved in autism, epigenetic anomalies in histone methylation patterns may contribute to the cerebellum neuroanatomical alterations, especially concerning Purkinje cells, observed in some individuals with ASD [for a review of cerebellar abnormalities reported in autism, see Ref. (184) and (185)]. The Purkinje cell maturation in the cerebellum is signaled by a normal downregulation of the engrailed-2 (EN-2) gene during late prenatal and early-postnatal development (186). James et al. (187) conducted a study on post-mortem cerebellar samples from 13 individuals with autism and found histone methylation modifications in the EN-2 promoter associated with increased EN-2 gene expression and EN-2 protein levels. These results suggest a postnatal persistence of EN-2 overexpression in some individuals with autism that may contribute to autism cerebellar abnormalities.

Another possible epigenetic mechanism that may underlie autism is RNA editing, a neurodevelopmentally regulated post-transcriptional mechanism responsible for producing mRNA molecules with sequence information not specifically encoded in the genome. More particularly, adenosine-to-inosine (A-to-I) RNA editing fine-tunes synaptic function (strength and duration) in response to environmental stimuli, affecting the transmission of all sensory stimuli to the CNS. A recent study (188) surveyed A-to-I RNA editing in autistic brains and found differential editing patterns and a dysfunctional form of the adenosine deaminase enzyme involved in RNA editing in post-mortem cerebella from individuals with autism.

Epigenetic mechanisms have been also reported in various genetic disorders associated with autism, including maternal 15q11–q13 duplication and several syndromes such as Fragile X, Rett, Down, Turner, Phelan-McDermid, Beckwith–Wiedemann, Williams–Beuren, CHARGE, Angelman, or Prader–Willi syndrome (see Tables 2 and 3). It is noteworthy that among the most commonly recurrent cytogenetic abnormalities associated with autism are duplications of sequences in a region on the proximal part of the long arm of chromosome 15, specifically the interval 15q11–q13. The behavioral phenotypes associated with 15q11–q13 defects show a parent-of-origin specific effect on phenotypic expression. More specifically, it is the maternally derived duplications that convey a high risk of autism (189–191). Similarly, paternal-specific deletion of multiple imprinted, paternally expressed genes on the 15q11–q13 region results in Prader–Willi syndrome, whereas maternal deletion of a single, imprinted, maternally expressed gene encoding a ubiquitin-protein ligase (UBE3A) on this same region gives rise to the Angelman syndrome phenotype. Phenotypic comparisons between Prader–Willi syndrome, Angelman syndrome, and maternal 15q11–q13 duplication reveal commonalities possibly related to a shared genetic basis. These phenotype overlaps concern common areas of cognitive impairment (or, more rarely, superior performance in discrete cognitive domains), motor stereotypies, motor coordination, seizures, language impairment, or behavioral manifestations such as compulsions or tantrums (see Table 2).

**Table 3:** **Epigenetic mechanisms and potential therapeutic targets in genetic disorders associated with autistic syndrome**.

Genetic disorder	Etiology	Epigenetic mechanisms	Potential therapeutic targets
CHARGE syndrome (192)	Mutations/deletions of CHD7 (8q12.1)	Chromatin remodeling	–
Maternal 15q11–q13 duplication (118, 119, 164)	Maternal duplications of 15q11–q13 region	Possible disruptions of normal parental homolog pairing, DNA methylation patterns, and gene expression patterns within 15q11–q13. However, according to some authors, such as Grafodatskaya et al. (164), the epigenetic mechanisms that mediate the development of ASD in association with 15q11–q13 duplication are still unidentified	–
Angelman syndrome (AS) (118, 193–195)	Mutation or deletion of the maternal UBE3A gene paternal UPD at 15q11–q13	Lack of expression of maternally expressed gene UBE3A in brain due to loss of DNA methylation at maternal allele of IC. The paternal allele of neuronal UBE3A, a ubiquitin-protein ligase (15q11), is epigenetically silenced	Topoisomerase inhibitors to restore UBE3A expression in neurons. Unsilence the imprinted paternal UBE3A allele with topotecan. Inhibition of paternal UBE3A antisense RNA transcript expression
Prader–Willi syndrome (PWS) (118, 196–198)	Paternal deletions, maternal UPD at 15q11–q13, deletions and epimutations of IC, translocations disrupting SNRPN	Gain of DNA methylation at paternal allele of IC blocks expression of paternally expressed genes from imprinted cluster at 15q11–q13	Folate fortification during at-risk pregnancies. DNMT inhibitors to release silenced maternal genes, combined with other epigenetic mechanisms for gene activation
Fragile X Syndrome (FXS) (199–203)	Inactivation of FMR1 gene (Xq28) due to a CGG expansion (>200 repeats) at the 5′ UTR region	DNA hypermethylation coupled with histone H3 and H4 tail deacetylation, histone H3-K9 methylation, and histone H3-K4 de-methylation	Pharmacologically restore FMR1 transcription through the use of the DNA demethylating agent 5′-aza-2′-deoxycytydine and/or inhibitors of histone deacetylases
Rett syndrome (119, 204–207)	MeCP2 (Xq28) loss-of-function missense/non-sense mutations or deletions	*De novo* (>90%) missense/non-sense mutations or deletions disrupt binding to methyl-CpG, causing anomalous gene transcription; disruptions in mRNA translation, histone methylation (H3K4, H3K9), and acetylation. Neurons mainly implicated, also astrocytes and microglia	Normalize MeCP2 levels by viral delivery of complementary DNA under native promoter to restore physiological levels of MeCP2; protein, microglial, bone marrow or other modes of MeCP2 transfer; folate supplements; aminoglycoside antibiotics for read-through suppression of non-sense mutations leading to full MeCP2 protein expression
Down syndrome (119, 208)	Trisomy for chromosome 21	Trisomy for chromosome 21 results in overexpression of genes leading to abnormal brain development	Suppress expression from one chromosome 21:
			– Whole chromosome silencing in trisomic neurons
			– Insertion of an inducible X-inactive specific transcript (XIST) into DYRK1A locus in induced pluripotent stem cells from a Down syndrome patient
			– XIST-mediated silencing reversed the deficits in neuronal proliferation and neural rosette formation
Turner syndrome (135, 164)	Most common monosomy for X chromosome	The syndrome is caused by dosage changes in genes of the X chromosome that escape X chromosome inactivation	–
		Potential imprinted gene on chromosome X	
Phelan–McDermid syndrome (PMDS) (209–211)	Inherited, *de novo* deletions (70% paternal) at 22q13 leading to loss of SHANK3	SHANK3 plays important roles in the formation, maturation, and maintenance of synapses. It controls dendritic spine function; binds Homer; influences neurexin–neuroligin coupling and controls glutamatergic, PI3-Kinase and mTOR signaling	Excitatory synaptic transmission in PMDS neurons can be corrected by restoring SHANK3 expression or by treating neurons with insulin-like growth factor 1 (IGF1). IGF1 treatment promotes formation of mature excitatory synapses that lack SHANK3 but contain PSD95 and *N*-methyl-d-aspartate (NMDA) receptors with fast deactivation kinetics
Beckwith–Wiedemann syndrome (BWS) (212–214)	Mutations, epimutations, UPD, and chromosome rearrangements at imprinted gene cluster on11p15.5	Overexpression of paternally expressed growth factor IGF2 due to gain of DNA methylation at paternal allele of IC1 and/or underexpression of maternally expressed growth suppressor CDKN1C due to loss of DNA methylation at maternal IC2	Overexpression of paternally expressed growth factor IGF2 due to gain of DNA methylation at paternal allele of IC1 and/or underexpression of maternally expressed growth suppressor CDKN1C due to loss of DNA methylation at maternal IC2
Williams–Beuren syndrome (WBS) (215)	Deletion of contiguous genes at 7q11.23 including GTF2I and GTF2IRD1 genes	Possible epigenetic alteration through GTF2I and GTF2IRD1 encode a family of transcription factors (TFII-I, BEN) critical in embryonic development. “Feed-forward model” of gene regulation to explain the specificity of promoter recognition by TFII-I	–

*CHD7, chromodomain helicase DNA binding protein 7; FMR1, fragile X mental retardation 1; IC, imprinting center; MECP2, methyl-CpG binding protein 2; SNRPN, small nuclear ribonucleoprotein polypeptide N; UBE3A, ubiquitin-protein ligase E3A; UPD, uniparental disomy. IGFI 1, insulin-like growth factor 1or 2 (IGF1 or IGF2); DNMT, DNA methyltransferases; CDKN1C, cyclin-dependent kinase inhibitor 1C*.

Given the number of genetic disorders associated with epigenetic etiologies comorbid with autism, it can be suggested that epigenetic mechanisms involving gene × environment interactions might be a common pathway for many cases of ASD. Furthermore, as underlined by Grafodatskaya et al. (164), new molecular technologies allowing the identification of critical epigenetic determinants open interesting perspectives, including therapeutic ones. It might serve in the development of innovative therapeutic strategies, as already applied with the treatment by the histone deactylase inhibitor valproate.

## Conclusion

Considering that environmental factors can modify the expression of genes and the potential role of epigenetic mechanisms in the development of ASD, it appears necessary to study in concert the genetic factors and the environmental factors in autism. Despite recent studies on environmental risk factors for ASD, no single and major environmental factor has been identified, suggesting that further research should study a combination of factors through an integrated approach including gene × environment interactions. This integrated clinico-biological approach takes into account the interactions between the genetic factors and the postnatal or prenatal environmental factors (psychosocial environment but also cytoplasmic and uterine environment with placental exchanges and hormonal effects). An allelic form can be present in the genotype without expressing itself if it is inhibited by signals mediating environmental, epigenetic, or genetic contributions. It is therefore important not to focus on the “genes of autism,” which implies determinism, but to study instead the effects of the genome integrated with the effects of the environment with possible plasticity. Epigenetic remodeling by environmental factors opens new perspectives for a better understanding, prevention and early therapeutic intervention of ASD. Finally, autism could be considered as the result of several genetic and environmental factors. Given this multifactorial etiology, it is probably through multivariate analyses and a multidisciplinary approach including the participation of biologists as well as clinicians that advances will be made.

## Conflict of Interest Statement

The authors declare that the research was conducted in the absence of any commercial or financial relationships that could be construed as a potential conflict of interest.
